# Removal of EpCAM-positive tumor cells from blood collected during major oncological surgery using the Catuvab device- a pilot study

**DOI:** 10.1186/s12871-021-01479-3

**Published:** 2021-10-29

**Authors:** Andreas Winter, Kai Zacharowski, Patrick Meybohm, Andreas Schnitzbauer, Peter Ruf, Claudia Kellermann, Horst Lindhofer

**Affiliations:** 1 Department of Anaesthesiology, Intensive Care Medicine and Pain Therapy, University Hospital Frankfurt, Theodor-Stern-Kai 7, 60590 Frankfurt am Main, Germany; 2 Department of Anaesthesiology, University Hospital Wuerzburg, Oberdürrbacher Straße 6, 97080 Wuerzburg, Germany; 3 Department of General and Visceral Surgery, University Hospital Frankfurt, Theodor-Stern-Kai 7, 60590 Frankfurt am Main, Germany; 4 Trion Research GmbH, Am Klopferspitz 19, 82152 Martinsried, Germany

**Keywords:** Intraoperative blood salvage, Leukocyte depletion filter, Tumor cell, EpCAM positive tumor, Catumaxomab

## Abstract

**Background:**

Intraoperative blood salvage (IBS) is regarded as an alternative to allogeneic blood transfusion excluding the risks associated with allogeneic blood. Currently, IBS is generally avoided in tumor surgeries due to concern for potential metastasis caused by residual tumor cells in the erythrocyte concentrate.

**Methods:**

The feasibility, efficacy and safety aspects of the new developed *Catuvab* procedure using the bispecific trifunctional antibody Catumaxomab was investigated in an ex-vivo pilot study in order to remove residual EpCAM positive tumor cells from the autologous erythrocyte concentrates (EC) from various cancer patients, generated by a IBS device.

**Results:**

Tumor cells in intraoperative blood were detected in 10 of 16 patient samples in the range of 69–2.6 × 10^5^ but no residual malignant cells in the final erythrocyte concentrates after *Catuvab* procedure. IL-6 and IL-8 as pro-inflammatory cytokines released during surgery, were lowered in mean 28-fold and 52-fold during the *Catuvab* procedure, respectively, whereas Catumaxomab antibody was detected in 8 of 16 of the final EC products at a considerable decreased and uncritical residual amount (37 ng in mean).

**Conclusion:**

The preliminary study results indicate efficacy and feasibility of the new medical device *Catuvab* allowing potentially the reinfusion of autologous erythrocyte concentrates (EC) produced by IBS device during oncological high blood loss surgery. An open-label, multicenter clinical study on the removal of EpCAM-positive tumor cells from blood collected during tumor surgery using the *Catuvab* device is initiated to validate these encouraging results.

## Background

Blood lost during major surgery is conventionally replaced using allogeneic blood transfusion with transfusion rates ranging from 35 to 77% [1, 2]. However, perioperative blood transfusion may be associated with increased risks of adverse surgical outcomes including mortality, wound infection, pulmonary and renal complications, sepsis and prolonged hospital stay [3]. Further risks are anaphylaxis, hemolytic reactions, transfusion-related acute lung injury, and viral infections [4, 5]. In addition to the potential risks, it should be noted that allogeneic blood has a lower oxygen-carrying capacity than autologous blood [6]. Preliminary data may suggest that allogeneic transfusion may also be an independent risk factor for cancer-specific mortality and overall mortality in cancer patients [6–8]. One explanation for adverse reactions is a general transient depression of the immune system following transfusion with blood products and transfusion-induced immunomodulation [9–11].

Reinfusion of blood collected in the surgical field, however, is an ancient idea successfully used by John Duncan in 1885 during leg amputation [12]. Some risks and disadvantages of using intraoperative blood salvage [IBS) like hemolysis or salvaged blood syndrome triggering the activation of the coagulation cascade, leading to increased vascular permeability are extremely rare side effects. The risk of disseminated intravascular coagulation due to reinfusion of free hemoglobin, denatured proteins, and micro-aggregates of platelets and leukocytes is regarded theoretical. In contrast to allogeneic red blood cell transfusion, no immunosuppression is expected for IBS treatment [8] resulting in cancer patients’ increased chance of relapse free survival and overall survival. IBS has no risk of transmitted disease, such as hepatitis or infection with cytomegalovirus or human immunodeficiency virus, due to test failures and untested unknown viruses. Thus, autologous blood salvage may even contribute to a higher rate of successful cancer treatments [13]. Autologous blood salvage is cost-effective compared to fully burdened allogeneic blood transfusion [3, 14]. The potential risk of infusing malignant cells into patients operated for cancer is the main concern about the safety of IBS. Hence, oncological surgery is still considered a contraindication to IBS in some guidelines [15, 16].

An effective and easy-to-implement method for removing tumor cells with metastasizing potential from blood collected during tumor surgery is still needed. A medical device (“*Catuvab*”) based on the selectivity of the monoclonal antibody Catumaxomab has been developed in order to eliminate this potential risk. Catumaxomab is a biologically engineered, intact, trifunctional bispecific anti-EpCAM x anti-CD3 antibody. EpCAM is a tumor-associated antigen that is overexpressed on most epithelial tumors (carcinomas) and therefore suitable for targeted anti-cancer treatment [17]. The anti-EpCAM binding arm of Catumaxomab [18] has been shown to bind EpCAM-positive tumor cells in vitro with high affinity and specificity [19–21].

The *Catuvab* procedure consists of Catumaxomab crosslinking EpCAM positive tumor cells with CD3 positive T-cells and Fc-gamma receptor positive immune cells, removal of these cell aggregates during a centrifugation step from EC and final removal of residual tumor cell containing cell aggregates during a final filtration step using a 40 μm leukocyte depletion filter (LDF).

This pilot ex-vivo study explored the feasibility, safety aspects and efficacy of *Catuvab* assessing the number of residual tumor cells, concentration of the pro-inflammatory cytokines IL-6, IL-8 and Catumaxomab antibody amount before, during and at the end of the *Catuvab* -procedure in the final product, the erythrocyte concentrate (EC) of 16 cancer patients with high probability to be EpCAM positive according to indication.

## Methods

### Patients

Sixteen consecutive cancer patients undergoing abdominal tumor resection at the University Hospital Frankfurt, Germany, were enrolled in this study (mean 66.8 years) between March 2019 and November 2019. Only tumor indications were selected known to be EpCAM-positive such as advanced colon cancer, cholangiocarcinoma, esophageal cancer; ovarian cancer; pancreatic cancer, bile duct cancer, rectum cancer and perihilar cholangiocellular carcinoma.

The clinical study protocol was approved in December 2018 by the Ethics Committee of the University Hospital Frankfurt (number 325/18) and each patient was provided with informed consent. All patients were aware of the procedure and were informed that the shed blood was collected for research purposes and would not be transfused back to them.

### *Catuvab* medical device and Catumaxomab

*Catuvab* was used in combination with mechanical auto-transfusion devices, which are part of the operating equipment and remove leukocytes from ECs as standard. The medical device *Catuvab* kit consists of the following components: Syringe containing 10 μg antibody (Catumaxomab) in 100 μl buffer (aseptically filled) with cannula, sterilized and sterile packed, Conformité Européenne (CE)-marked; 6R vial with 5.7 mL 0.9% NaCl solution (aseptically filled); 2 pieces of 2 mL syringe with 100 μL graduation with cannula (21G × 1½”, 40 mm), sterilized and sterile packed, CE-marked two sterile LDFs, pore size 40 μm, with silicone hose and standardized connection. The manufacturer of the investigational medical device *Catuvab* is LINDIS Blood Care GmbH, Neuendorfstr. 20b, Henningsdorf, Germany. Catumaxomab is a biologically engineered, intact, trifunctional bispecific EpCAM x CD3 binding monoclonal antibody consisting of a mouse immunoglobulin G (IgG)2a chain and a rat IgG2b chain [19, 22, 23].

EpCAM is strongly expressed in squamous cell carcinomas derived from epithelial tissue and can be found in various tumors of epithelial original (gastric carcinoma, ovarian carcinoma, pancreatic carcinoma, colon / rectal carcinoma, non-small cell lung cancer, or peritoneal carcinomatosis) [23–25]. In the past, Catumaxomab has been developed as a targeted therapy for intraperitoneal treatment of malignant ascites and epithelial cancers expressing the EpCAM antigen (e.g. bladder, ovarian, pancreatic, lung and gastric cancer). In the lead indication treatment of malignant ascites due to epithelial cancers, the European Medicines Agency (EMA) approved Catumaxomab in 2009. For commercial reasons, the product was withdrawn in 2017.

The primary mode of action of Catumaxomab in the *Catuvab* device consists of the physical aggregation of tumor cells and lymphocytes/accessory cells and the subsequent removal of the cell aggregates by centrifugation and filtration as part of a machine autotransfusion. Simultaneous binding ex vivo of the antibody to lymphocytes in the patient’s intraoperative blood (via the CD3-specific region of the antibody), tumor cells and FcγR-positive accessory immune cells ultimately leads to the formation of larger cell aggregates.

### Technical procedure and blood sampling

**Fig. 1:**
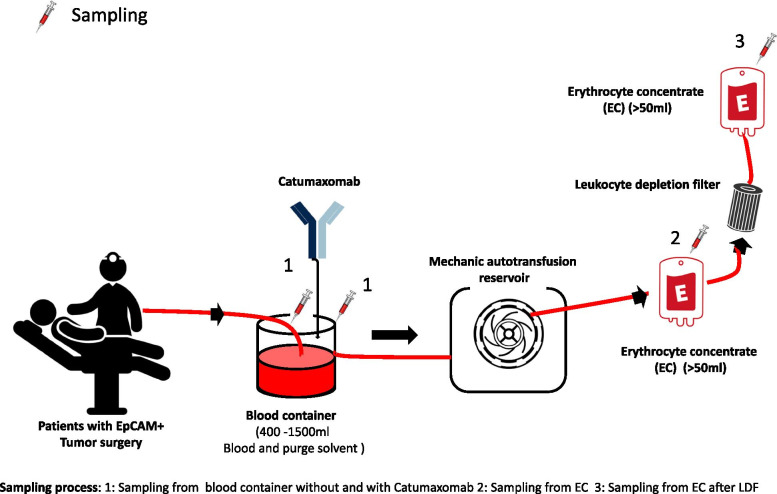
Sampling procedure of the Catuvab pilot study

Three parameters were investigated in intraoperative blood, during processing and in the final product after filtration with a leukocyte depletion filter (LDF) (Fig. 1):

1. Detection and quantification of EpCAM positive tumor cells in patient blood, the EC and EC after LDF
2. Detection and quantification of cytokines IL-6, IL-8 in the reservoir, in EC and in EC after LDF (probe sampling 1, 2 and 3)
3. Detection and quantification of Catumaxomab in EC after LDF (probe sampling 3).

Blood and purge solvent accumulated during surgery was collected in a reservoir containing a bone splinter filter (ATR 120 reservoir, Fresenius Kabi, Fig. 1). The blood and purge solvent mixture collected in the reservoir was centrifuged and washed using a IBS machine (C.A.T.S.+, Fresenius Kabi, AT3 Autotransfusionsset, Fresenius Kabi), resulting in an erythrocyte concentrate (EC). The erythrocyte concentrate (EC) was filtered using a 40 μm Leukocyte depletion filter (LDF, RS1, Haemonetics) (LDF sample 3). All samples for analysis were extracted via an output connection/an outlet and collected in sterile tubes. The samples collected for antibody analysis were frozen within 1-2 h (− 20 °C). The samples were sent immediately to Trion Research GmbH. The blood samples for cytokine analysis were centrifuged, the supernatant (plasma) collected, frozen within 1-2 h (− 20 °C). The samples were sent immediately to the analysis laboratory on dry ice.

Catumaxomab was first diluted and a defined amount of the diluted antibody (2,5 μg or 5 μg antibody) was supplied to the blood mixture via a port on the reservoir, using a syringe. Antibodies were distributed within intraoperative blood and aggregates of tumor and immune cells developed within approximately 30 min. During the usual washing and concentration process of the IBS, cell aggregates with a relatively lower density could be separated from the red blood cells in the total mixture by centrifugation. A second filtration step (LDF filter of *Catuvab*) removed any remaining cell aggregates.

### Detection and quantification of EpCAM positive tumor cells, Catumaxomab and IL-6, IL-8

Detection and quantification of EpCAM positive tumor cells were performed by immunofluorescence staining using the tumor marker EpCAM and cytokeratin. Density gradient centrifugation applying Ficoll-Paque was used as separation medium for lymphocytes and tumor cells that were stained after centrifugation on cytospin preparations. These cytospins were analyzed for the presence of EpCAM-positive tumor cells using the antibody BER-EP4. Quantification of tumor cells was performed by immunofluorescence microscopy with integrated digital image analysis (Applied Imaging) [26].

### Detection and quantification of EpCAM binding Catumaxomab in EC before and after LDF

Catumaxomab concentrations were measured by an established two-site ELISA. Briefly, catumaxomab was captured by an anti-rat IgG light chain-specific antibody (LA1B12, TRION Research, Munich, Germany). Bound catumaxomab was then detected via an anti-mouse.

IgG2a-specific biotin-labeled detection antibody (BD Pharmingen, San Diego, CA). Then, streptavidin-b-galactosidase and its corresponding substrate, chlorphenolred-β-D-galactopyranosid (Roche Diagnostics, Mannheim, Germany), were added, and the colorimetric reaction was measured at 570 nm. Catumaxomab concentrations were calculated by interpolation on a standard curve. The lower limit of quantification (LLOQ) of the assay was determined to be 125 pg ml-1; the upper limit of quantification was 4000 pg ml-1. All samples were diluted 1:2 before measuring in duplicate [19, 26].

### Detection and quantification of cytokines IL-6, IL-8 in the reservoir, in EC and in EC after LDF

As Catumaxomab is well known to activate different types of immune cells, the aim of the measurements was to determine a potential increase of proinflammatory cytokines during the *Catuvab* procedure [27]. Samples were sent to Synlab MVZ laboratory (Munich, Germany) and cytokines were determined by Luminex® Corporation Multiplex technology using magnetic microsphere beads. The Multiplex ELISA is based on unique fluorescent signature coated microbeads binding specific cytokines which are subsequent measured by laser technology.

### Statistics

Due to the exploratory character and the low number of patients of this pilot study only descriptive statistics using mean values were performed.

## Results

### Intraoperative blood volumes

The *Catuvab* procedure was applied extra-corporally to the intraoperative blood from a total of 16 subjects during surgery. The EC was not re-transfused. For 1 subject (No. 9), obviously no antibody was applied (antibody not detectable for unknown reason in the reservoir), so this subject and related samples were excluded from Catumaxomab antibody amount calculations. The volume of the intraoperative blood mixture (blood and dilution fluid) ranged from 500 ml up to 2800 mL and the volume of the added dilutive solution during surgery from 0 up to 2500 ml. The volume of undiluted intraoperative blood ranged between 300 and 1300 ml for the group of patient samples treated with 2.5 μg Catumaxomab to generate tumor cell aggregates. For the group of patient samples treated with 5 μg, the volume of undiluted (and diluted) intraoperative blood ranged from 300 ml (1400 ml) up to 2550 ml (2600 ml).

### Detected tumor cells

In 10 out of the 16 intraoperative blood samples (Reservoir, EC) (63%), EpCAM-positive tumor cells were detected in the different *Catuvab* procedure steps. The number of EpCAM-positive tumor cells ranged from 69 in an EC sample (before filtration (LDF)) to 263,076 in the Reservoir. Finally, no tumor cells were found following the last purification step 3 (leukocytes filtration) in the final EC product (Table 1).

**Table 1:** Results of Measurement of EpCAM-Positive Tumor Cells

Subject number	1	2	3	4	5	6	7	8	9	10	11	12	13	14	15	16
**Cancer**	**Esoph.**	**CCA**	**Rectal**	**Esoph.**	**CCA**	**Pancr.**	**Gist**	**Colon**	**CCA**	**Coecum** **+Ovar.**	**Pancr.**	**Pancr.**	**Bile duct**	**Rectal**	**Bile duct**	**pCCC**
**Surgery blood (mL)** **Dilution fluid (mL)**	**1400** **550**	**500** **0**	**2800** **2500**	**1600** **1000**	**1100** **500**	**1600** **500**	**800** **300**	**950** **300**	**1000** **700**	**2200** **200 + 800**	**800** **150**	**1700** **1400**	**1661** **900**	**2600** **50**	**1400** **100**	**800** **200**
Total number of tumor cells in IBS Reservoir^a^	7600	0	263,076	0	14,322	108,984	108	0	0	101,525	15,529	0	0	0	0	0
Total number of tumor cells in EC^b^	0	192	6835	0	2756	0	0	0	0	4163	1886	69	0	0	419	0
Total number of tumor cells in EC after filtration (LDF)^c^	0	0	0	0	0	0	0	0	0	0	0	0	0	0	0	0

The numbers of tumor cell in the reservoir and ECs were assessed in test samples (20 ml) and calculated to the total volume. In the EC following filtration, the total volume was consumed for tumor cell assessment

CCA: cholangiocarcinoma; EC: erythrocyte concentrate; EpCAM: epithelial cell adhesion molecule; Esoph.: esophageal cancer; LDF: leukocyte depletion filter; Ovar.: ovarian cancer; Pancr.: pancreatic cancer; pCCC: perihilar cholangiocellular carcinoma

^a^ IBS Reservoir: Blood and purge solvent accumulated during surgery was collected in a reservoir containing a bone splinter filter (IBS/ Reservoir sample)

^b^ EC: Blood and purge solvent mix collected in the reservoir was centrifuged and washed using a IBS machine, resulting in an EC (EC sample)

^c^ LDF: EC was filtered using a 40 μM LDF (Haemonetics), resulting in an EC containing almost no white blood cells (LDF sample)

### Measured cytokines

Proinflammatory cytokines (IL-6 and IL-8) were measured in the reservoir before administration of the crosslinking antibody Catumaxomab as well as in the EC before and after filtration (final product) of the 15 antibody receiving subjects. The values for IL-6 and for IL-8 (given in ***bold***) in intraoperative blood ranged from below the level of quantification (BLQ = 9.8 pg/mL; ***3.1 pg/mL***) to 2633 pg/mL (***518 pg/mL***) in the reservoir before the administration of catumaxomab (mean: 662 pg/mL; ***129 pg/mL***). After administration of catumaxomab, values ranged from 25 to 1070 pg/mL (***10 to 1046 pg/mL***) (mean: 339 pg/mL; ***323 pg/mL***) in the EC (before filtration). After filtration, the values in the EC ranged from BLQ to 2488 pg/mL (***BLQ to 139 pg/mL***) (mean: 516 pg/mL; ***46 pg/mL***) (Table 2).

**Table 2:** Results of Measurement of Cytokines Interleukin-6 and Interleukin -8

Subject number		1	2	3	4	5	6	7	8	9	10	11	12	13	14	15	16
Cancer		Esoph.	CCA	Rectal	Esoph.	CCA	Pancr.	Gist	Colon	CCA	Coecum +Ovar.	Pancr.	Pancr.	Bile duct	Rectal	Bile duct	pCCC
Surgery blood (mL) Dilution fluid (mL)		1,400 550	500 0	2,800 2,500	1,600 1,000	1,100 500	1,600 500	800 300	950 300	1,000 700	2,200 200+800	800 150	1,700 1,400	1,661 900	2,600 50	1,400 100	800 200
IL-6/ IL-8 (pg/mL)	Reservoir	BLQ/ 6	BLQ/ 92	2633/ 322	90/ 16	257/ 518	121/ 25	163/ 75	23/ 17	BLQ/ 9	2617/ 76	688/ 232	43/ 108	284/ 100	2271/ 157	76/ 56	244/ 114
	EC	1054/ 1032	25/ 103	219/ 1046	727/ 569	221/ 199	91/ 25	267/ 102	17/ 10	-	1070/ 383	49/ 353	243/ 97	317/ 138	191/ 180	264/ 284	29/ 821
	LDF	408/ 24	55/ 112	236/ 139	780/ 13	317/ 58	114/ 9	260/ 24	22/ BLQ	BLQ/ BLQ	2488/ 72	20/ 5	492/ 86	184/ 17	554/ 11	1289/ 67	BLQ/ 21
IL-6/ IL-8 (total, ng)	Reservoir	14.7/ 8.4	5.2/ 46	7372/ 901	144/ 26	283/ 570	194/ 40	130/ 60	22/ 16	11/ 09	5757/ 167	550/ 186	73/ 184	472/ 166	5904/ 408	106/ 78	195/ 91
	LDF	43.6/ 2.6	2.3/ 4.6	27/ 16	49/ 0.8	20/ 4	17/ 1	17/ 2	1/ 0.1	0.4/ 0.1	264/ 8	1/ 0.2	33/ 6	8/ 0.7	177/ 4	77/ 4	2/ 4

BLQ: below the limit of quantification of 9.8 pg/mL for IL-6 and 3.1 pg/mL for IL-8

CCA: cholangiocarcinoma; EC: erythrocyte concentrate; Esoph.: esophageal cancer; IL: interleukin; LDF: leukocyte depletion filter; Ovar.: ovarian cancer; Pancr.: pancreatic cancer; pCCC: perihilar cholangiocellular carcinoma

As during the *Catuvab*- procedure the volumes of intraoperative blood decreased from, e.g., 2800 mL in the reservoir (mean: 1461 mL) to 40 mL in the EC (mean: 99 mL), the total amount of cytokines (IL-6 and IL-8) was also calculated in the reservoir and the EC (after filtration) to assess (i) the reduction in total amount of cytokines during the procedure, and (ii) amount of cytokines that would potentially be reinfused in the patient. The total amount of IL-6 (IL-8 in ***bold***) ranged from 5.2 ng (***8.4 ng***) to 7372 ng (***901 ng***) (mean: 1502 ng; ***204 ng***) in collected intraoperative blood in the reservoir. After completion of the *Catuvab* procedure, the values of IL-6 (IL-8 in ***bold***) ranged from 1 ng (***0.1 ng***) to 264 ng (***16 ng***) (mean: 53 ng; ***3.9 ng***) in the EC (final product) (Table 2). For the mean values, a 28-fold (***52-fold***) reduction was observed in the total amount of cytokines in the final EC when compared to unprocessed collected intraoperative blood in the reservoir.

### Residual antibodies

An important safety aspect of the medical device is the absence of antibody or very low residual antibody in the final product, which is intended to be re-transfused to the subject. During the exploratory pilot study, different ways to apply the antibody were tested. For the first 10 subjects, the way of antibody application was not clearly defined, and in some cases the antibody was applied first in the reservoir before the collection of intraoperative blood was started. This was the case for Subjects 2 and 7, who exhibited a relatively high concentration of antibody (1694 pg/mL and 1026 pg/mL, respectively) following the application of 2.5 μg antibody (Table 3). To improve the procedure, in the next 5 subjects (No. 11–16), the antibody was applied only after a minimum of 350 mL intraoperative blood volume was collected in the reservoir, enabling an improved interaction and binding of the antibody with immune cells and EpCAM- positive tumor cells. This change in the experimental setting probably resulted in a decreased antibody concentration below the limit of quantification (BLQ) in Subjects 11 and 16, and to 160 pg/mL in Subject 15 (Table 3). Blood volumes exceeding > 1500 mL were collected from patient 12 and 13 and further 2.5 μg antibody was applied to the reservoir. This resulted in higher concentrations of antibody in the EC (888 pg/mL and 628 pg/mL, respectively). These results suggested to limit intraoperative blood volume to 1500 mL per IBS cycle (a second cycle could be started if the total amount of intraoperative blood was ≥1500 mL). After establishment of both improvements regarding antibody application in the reservoir starting from patient 14–16, the total amounts of antibody found in the final EC ranged from BLQ (≤125 pg/mL) up to 9 ng (Table 3).

**Table 3:** Results of Antibody Measurement

Subject number	1	2	3	4	5	6	7	8	9	10	11	12	13	14	15	16
**Cancer**	**Esoph.**	**CCA**	**Rectal**	**Esoph.**	**CCA**	**Pancr.**	**Gist**	**Colon**	**CCA**	**Coecum** **+Ovar.**	**Pancr.**	**Pancr.**	**Bile duct**	**Rectal**	**Bile duct**	**pCCC**
**Surgery blood (mL)** **Dilution fluid (mL)**	**1400** **550**	**500** **0**	**2800** **2500**	**1600** **1000**	**1100** **500**	**1600** **500**	**800** **300**	**950** **300**	**1000** **700**	**2200** **200 + 800**	**800** **150**	**1700** **1400**	**1661** **900**	**2600** **50**	**1400** **100**	**800** **200**
EC after LDF, residual Ab (pg/mL)	BLQ	1694	BLQ	BLQ	205.0	BLQ	1026	491.0	ND	252.0	BLQ	888.0	628.0	BLQ	160.0	BLQ
Ab total (ng)	BLQ	69.0	BLQ	BLQ	13.0	BLQ	68.0	22.0	BLQ	27.0	BLQ	59.0	26.0	BLQ	9.0	BLQ
Vol. EC (mL)	142.0	76.0	150.0	98.0	97.0	187.0	101.0	80.0	70.0	141.0	75.0	101.0	77.0	373.0	133.0	–
Vol. EC post LDF (mL)	107.0	41.0	115.0	63.0	62.0	152.0	66.0	45.0	35.0	106.0	40.0	66.0	42.0	320.0	60.0	200.0
Amount of applied antibody (μg)	5.0	2.5	2.5	2.5	2.5	5.0	2.5	2.5	–	5.0	2.5	5.0	5.0	5.0	2.5	2.5

*BLQ* Below the limit of quantification of 125 pg/mL

*Ab* Antibody, *CCA* cholangiocarcinoma, *EC* Erythrocyte concentrate, *Esoph*. Esophageal cancer, *LDF* Leukocyte depletion filter, *ND* No antibody detectable in the reservoir, *Ovar*. Ovarian cancer, *Pancr*. Pancreatic cancer, *pCCC* Perihilar cholangiocellular carcinoma, *Vol*. Volume

For the upcoming multicenter REMOVE study with the reinfusion of *Catuvab*- treated EC the antibody will be added after a minimum (400 mL) intraoperative blood collection and instituting a maximum of 1500 mL intraoperative blood per IBS cycle.

## Discussion

The spreading of tumor cells during surgery can originate from tumor cells left at the resection line, inadvertent rupture of the tumor, prior presence of tumor cells in the peritoneal cavity, or intraoperative release into the blood by pressurization, but unlikely from circulating tumor cells [28, 29]. Several studies have demonstrated that tumor cells are commonly detected in red cell concentrates in autologous reinfusion bags in 62–90% of cases and these cells demonstrate proliferation capacity, invasiveness, and tumorigenicity [28, 30, 31]. Hansen et al. [28] suggested that tumor cells identified from surgical fields are different from those circulating in the peripheral blood, as both the detection frequency and number of tumor cells are much higher in surgical fields than those in the circulation at the end of surgery. It was also found that an IBS itself was not sufficient for removing tumor cells in most cases [30].

To handle this problem, leukocyte depletion filters (LDFs) have been tested in spine-cancer patients and in a variety of urologic malignancies, including prostate, urothelial, renal and liver cancer [32–35].

Kumar et al. [36] showed that IBS using LDFs can effectively eliminate tumor cells from salvaged blood in spinal tumor surgery in 8 of 11 tested subjects. The mechanisms for the entrapment of tumor cells by LDF is likely a combination of mechanical sieving and unspecific biological adhesion processes [37]. However, whether tumor cells are completely filtered in clinical settings and whether LDFs eliminate the risk of tumor cell metastasis, remains unknown. Thus, regulatory guidelines e.g. in Germany (Querschnittsleitlinie der Bundesärztekammer 2020, www.baek.de) prohibit the retransfusion of autologous ECs from intraoperative blood gained during cancer surgery.

Wu et al. [38] conducted a meta-analysis to evaluate the oncological safety of pure IBS versus allogeneic blood transfusion in surgery of malignant disease. IBS with LDF was reported to be comparable to allogeneic blood regarding tumor recurrence rate, regardless of the effect-cost ratio or the efficacy in tumor surgery. However, published data suggest that the capability of an LDF to filter tumor cells is load limited. Thus, when the number of tumor cells is too high (≥2 × 10^3^), the filter will fail to remove tumor cells completely, so that the risk of distant spread of the tumor still remains [31, 37, 39].

As an alternative, the irradiation of blood prior to its reinfusion has also been proposed. Blood irradiation ensures a 10 to 12 log reduction in the number of tumor cells, which is considered sufficient to eliminate all tumor cells without impairing the function of red blood cells [31]. Besides that, irradiation also damages the DNA of malignant cells, reducing their multiplication capacity. Irradiation treatments require special large-scale radiation equipment as well as strict radiation protection management. Most of medical institutions worldwide do not have such conditions, and irradiation treatment cannot usually be completed in the operating room, which reasonably limits its wide clinical implementation.

An effective and easy-to-implement method for removing EpCAM positive tumor cells with metastasizing potential [40, 41] from blood collected during tumor surgery was invented based on the selectivity of the monoclonal anti-EpCAM antibody Catumaxomab. EpCAM is expressed by a broad spectrum of epithelial solid cancer types in the range of > 90% (as e.g. ovarian-, gastric-, colonic-, pancreas-, bladder-, prostate-, endometrial- and non- small cell lung cancer) [24, 25] and Catumaxomab is able to bind even at a very low EpCAM expression levels due to its high affinity and cell binding potential [20] making *Catuvab* applicable for a broad spectrum of solid cancer surgeries.

Here, we have to discuss the role of EpCAM negative tumor cells, which could potentially escape the *Catuvab* procedure. In this context, three conditions have to be considered. Firstly, only cancer indications which express EpCAM in > 90% of cases should be considered. Secondly, also EpCAM negative tumor cells will be reduced up to 90% due to centrifugation and filtration steps independent of the binding and crosslinking ability of the trifunctional antibody (filter characteristics). Thirdly, several publications have demonstrated a higher metastasizing potential for EpCAM positive carcinoma cells compared to EpCAM negative tumor cells [40, 41]. Taken together, it seems that the risk /benefit ratio using *Catuvab* regarding the potential contamination with residual EpCAM negative tumor cells might be acceptable, but needs further clinical evaluation.

This pilot study showed that it is feasible to implement *Catuvab* procedure easily in the blood collection procedure. Even at a high tumor cell level in intraoperative blood (e.g. > 2.6 × 10^5^) it was possible to eliminate these cells after the final LDF filtration step. IL-6 and IL-8 amounts could be markedly reduced in mean 28-fold and 52-fold respectively indicating cytokine-wash out effects of the procedure. The mean values of the total amount of IL-6 and IL-8 in the final product are considered uncritical given the approximately 2000- to 3000-fold dilution in the subject’s body. This interpretation is based on the calculation that patients body blood volume ranges in average between 5 and 7 l containing about 2–3 l of plasma. Even the measured peak value of 264 ng of IL-6 would not lead to critical values in this scenario. Thus, safety aspects regarding pro-inflammatory cytokines should not be an issue for the *Catuvab* procedure.

Residual Catumaxomab antibody was detected in 8 of 16 of the final EC products at a decreased amount (37 ng in mean) which is considered non-critical regarding the LD_50_ > 5.0 mg/kg in mice [42], no toxicity signs up to and 300 μg/kg in cynomolgus monkeys [43], the MTD of 7 μg confirmed in clinical trial [44] exceeding the residual drug in the EC by magnitudes of order as well as the general clinical experience [26].

## Conclusion

As auto-transfusion devices itself are not sufficient for removing tumor cells [28, 29] and additional measures such as the application of LDF markedly reduced the risk for reintroduction of tumor cells, but failed in the presence of a high tumor load in salvaged blood [37], a residual risk of contamination remains. Despite to the low number of patients, the results of this ex vivo study indicate a complete removal of EpCAM positive tumor cells, which has to be validated in a clinical study. The primary objective of this upcoming REMOVE study is to demonstrate that the *Catuvab* device utilized during IBS procedures (including centrifugation step and leukocyte depletion filter) depletes EpCAM-positive tumor cells effectively in autologous blood retransfusion.

## Data Availability

The datasets analysed are available from the corresponding author upon reasonable request. For data request please contact Andreas Winter, University Hospital Frankfurt, Germany (andreas.winter@kgu.de).

## Abbreviations

Ab: Antibody
BLQ: Below the limit of quantification
CCA: Cholangiocarcinoma
EC: Erythrocyte concentrate
EpCAM: Epithelial cell adhesion molecule
Esoph: Esophageal cancer
IBS: Intraoperative blood salvage
IL: Interleukin
LDF: Leukocyte depletion filter
ND: No antibody detectable in the reservoir
Ovar: Ovarian cancer;
Pancr: Pancreatic cancer;
pCCC: Perihilar cholangiocellular carcinoma
Vol: Volume
